# Hearing loss among elderly people and access to hearing aids: a cross-sectional study from a rural area in Germany

**DOI:** 10.1007/s00405-021-06799-1

**Published:** 2021-04-18

**Authors:** Birgit Didczuneit-Sandhop, Katarzyna Jóźwiak, Manja Jolie, Josefine Holdys, Michael Hauptmann

**Affiliations:** 1 Department of Otorhinolaryngology, Head and Neck Surgery, University Hospital Brandenburg, Brandenburg Medical School Theodor Fontane, Brandenburg a.d. Havel, Germany; 2 Institute of Biostatistics and Registry Research, Brandenburg Medical School Theodor Fontane, Neuruppin, Germany; 3 Faculty of Health Sciences Brandenburg, Brandenburg Medical School Theodor Fontane, Neuruppin, Germany

**Keywords:** Hearing loss, Rural, Audiometry, Hearing aid, Health services

## Abstract

**Purpose:**

Hearing loss is common and associated with reduced quality of life, particularly among elderly people. However, many patients do not use hearing aids. We evaluated the use of hearing aids among people with hearing loss by health services availability near their residence in a rural area in the state of Brandenburg, Germany.

**Methods:**

Audiometry was performed in a convenience sample of subjects in ten towns and hearing loss was determined, defined as a threshold of ≥ 30 dB in at least one ear and at least one of the frequencies 0.5, 1.0, 2.0 and 4.0 kHz. For each participant, age and gender were collected and whether or not hearing aids were available.

**Results:**

Among 186 persons with an average age of 74 years (interquartile range 71–81), 97% had hearing loss [95% confidence interval (CI) 95–100]. Among 121 patients with hearing loss who reported whether or not they have a hearing aid, 93 had no hearing aid (77%, 95% CI 69–84). The proportion of hearing-impaired persons who do not have a hearing aid significantly increased with the absence of a hearing aid specialist or ear nose throat (ENT) physician or both in the town where the tests were performed (*p* trend = 0.001).

**Conclusion:**

Hearing loss is common among elderly people in the study area and many people in rural areas in Germany may not be properly supplied with hearing aids due to lack of hearing aid specialists and/or ENT physicians close to their residence. Interventions to improve this situation are urgently required.

## Introduction

Over 5% of the world’s population suffer from hearing impairment defined as hearing loss greater than 40 dB in the better hearing ear for adults and greater than 30 dB for children. It is estimated that by 2050 over 900 million people—or one in ten—will have disabling hearing loss [1]. The most common cause of hearing impairment in elderly people is age-related hearing loss or presbyacusis. Other causes for hearing loss in adulthood are chronic otitis media and acoustic trauma [2].

Hearing loss can have profound effects on health, since it is associated with poor cognitive performance [3], increased risk of falls [4], acceleration of dementia [5], social isolation and psychiatric disorders including depression, anxiety and schizophrenia [6–8]. Therefore, hearing loss is now the fourth (men) and seventh (women) leading cause of years lived with disability in the Global Burden of Disease Study [9], higher than many other diseases such as stroke, falls and dementia.

Presbyacusis is underdiagnosed and undertreated in Germany. A recent systematic review on hearing loss in Germany [10] identified only six available cross-sectional studies [11–16]. The observed proportion ranged between 16 and 25% and varied by age, study design and definition of hearing loss. Two studies indicate that the majority of patients are not properly supplied with hearing aids [12, 15]. An urgent need for advanced studies of the determinants of and interventions against untreated hearing impairment was expressed [17].

Definitions of hearing loss are very heterogeneous and use different thresholds (dB) for at least one or the average of several frequencies (commonly 0.5, 1, 2 and 4 kHz) in at least one ear or in the better hearing ear [10]. According to the World Health Organization, disabling hearing loss is defined as an average threshold exceeding 40 dB for the frequencies 0.5, 1, 2 and 4 kHz in the better hearing ear. Here, we apply the definition used by German health insurances as an indication for a hearing aid, which is a threshold of ≥ 30 dB for at least one of the frequencies 0.5, 1, 2 and 4 kHz in at least one ear.

Potential inequalities of health and health care in rural areas are of great concern not only in developing but also in industrialized countries [18], and about 30% of the area of European countries is considered thinly populated, i.e., < 100 inhabitants per km^2^ [19]. Indeed, Koster et al. [20] showed that in Western-European countries (including France, UK, Norway, Netherlands and Germany), several health indicators suggested better health among people living in urban areas compared to the total population. Investigating the rural component of diseases of high global burden, such as hearing loss, is therefore, key to understand and improve the public’s health.

We report the results of a small pilot study on the extent and determinants of untreated hearing impairment in a rural area of Germany, to prepare a comprehensive study from which interventions can be conceived.

## Methods

### Study population

Ten towns located around the city of Brandenburg a. d. Havel were visited between May 15 and 21, 2019 by a specifically equipped vehicle for performing audiometry (“Cochlea-Mobil”). A free hearing test was offered to anybody interested, irrespective of any exclusion criteria. The opportunity of free hearing tests was widely advertised beforehand in newspapers and posters in the cities.

### Hearing level measurements

The vehicle was noise-insulated and equipped with a micro-audiometer (MA33, Maico). Audiometry was conducted by an ENT physician from the hospital in Brandenburg a. d. Havel. Participants were considered to have hearing loss, if the threshold for at least one of the frequencies 0.5, 1.0, 2.0 and 4.0 kHz was ≥ 30 dB for at least one ear. Only air conduction was measured. The results were communicated to the participant and self-reported information on age, gender and availability of hearing aids was documented.

### Statistical analysis

We evaluated whether the participants with and without hearing loss differed by town, age, and gender using Fisher’s exact test (town, gender) and the Mann–Whitney *U* test (age). We calculated percentages of persons with no hearing devices among the persons with hearing loss. We tested whether the proportion of patients with untreated hearing impairment was associated with the absence of health professionals (hearing aid specialist, ENT physician or both) in the town using a linear-by-linear trend test for ordered proportions (order: no hearing aid specialist and no ENT physician, hearing aid specialist or ENT physician, hearing aid specialist and ENT physician). Null hypotheses were rejected when the two-sided *p* value was 5% or less. The statistical software STATA/SE 16 was used (Stata Corp 2019).

## Results

Audiometry was conducted for 186 persons and information on age (average 74.4 years; range 37–93) and gender (55.7% female) was provided by 148 and 149 persons, respectively. Hearing loss was detected in 181 participants [97%, 95% confidence interval (CI) 95–100] (Table 1). The average threshold for the right (left) ear was 47.5 dB (48.0 dB) across frequencies 0.5, 1.0, 2.0 and 4.0 kHz.

**Table 1:** Overview of study population

	Hearing loss^a^		Total *N*	*p* value^b^
	Yes *N* (%)	No *N* (%)		
Place of hearing measurement				
Bad Belzig	15 (94)	1 (6)	16	0.307
Lehnin/Brück/Treuenbrietzen	28 (97)	1 (3)	29	
Luckenwalde/Jüterborg	33 (94)	2 (6)	35	
Premnitz/Genthin	52 (100)	0 (0)	52	
Rathenow	30 (97)	1 (3)	31	
Ziesar	23 (100)	0 (0)	23	
Gender				
Female	81 (98)	2 (2)	83	0.999
Male	64 (97)	2 (3)	66	
Unknown	36 (97)	1 (3)	37	
Age at hearing measurement				
Mean (IQR), yrs	75.1 (71, 81)	50.5 (40.5, 60.5)	74.4 (70.5, 81)	0.003
Unknown	37 (97)	1 (3)	38	
Hearing device				
Yes	28 (100)	0 (0)	28	0.999
No	93 (98)	2 (2)	95	
Unknown	60 (95)	3 (5)	63	
Total	181 (97)	5 (3)	186	

^a^Hearing loss was defined as an average threshold of 30 dB or more for at least one of the frequencies 0.5, 1.0, 2.0 and 4 kHz in at least one ear

^b^Fisher’s exact test for place, gender and hearing device, Mann–Whitney *U* test for age among subjects with known values

The proportion of patients with hearing loss was similar across towns (*p*  = 0.307): 94% in Bad Belzig, 97% in Lehnin/Brück and Treuenbrietzen, 94% in Luckenwalde/Jüterbog, 100% in Premnitz and Genthin, 97% in Rathenow and 100% in Ziesar (Table 1).

For 121 patients with hearing loss, it is known whether they have a hearing aid. Of those, 93 had no hearing aid (77%, 95% CI 69–84). The proportion of patients with hearing loss who do not have a hearing aid is increasing from 45 patients (67%) in towns with an ENT physician and a hearing aid technician (Bad Belzig, Premnitz/Genthin) to 24 (80%) in towns with only a hearing aid technician (Rathenow) to 24 patients (100%) in towns with neither profession (Ziesar and Lehnin/Brück/Treuenbrietzen), which is a significant trend (*p* = 0.001) (Table 2).

**Table 2:** Frequency of patients with and without a hearing aid by the presence of a hearing aid technician or ENT physician at the place of residence among patients with hearing loss and available information whether they had a hearing aid (*N* = 121)

Available at place of residence	Hearing aid		Total *N* (%)	Trend *P*^a^
	No *N* (%)	Yes *N* (%)		
Hearing aid technician and ENT physician^b^	45 (67)	22 (33)	67	0.001
Hearing aid technician, no ENT physician^c^	24 (80)	6 (20)	30	
No hearing aid technician and no ENT physician^d^	24 (100)	0 (0)	24	
Total	93 (77)	28 (23)	121	

^a^Linear-by-linear test of the null hypothesis of no increase or decrease of the proportion of patients with hearing aids by availability of hearing aid technician and/or ENT physician at the place of residence

^b^Towns were Bad Belzig, Premnitz/Genthin

^c^Town was Rathenow

^d^Towns were Ziesar, Lehnin/Brück/Treuenbrietzen

Data per ear by hearing thresholds above 60, 70 and 80 dB at 0.5, 1.0, 2.0, and 4.0 kHz were available for 181 patients and 354 ears (for 8 patients, values were known for only 1 ear). An average hearing loss exceeding 60 (70, 80) dB was observed in 87 (48, 22) ears (24.6%, 13.6%, 6.2%), including 26 (13, 9) ears without a hearing aid.

## Discussion

In a cross-sectional convenience sample of mostly elderly people from ten rural towns in the German state of Brandenburg, we observed a high proportion of patients with hearing loss. The majority of patients with hearing loss did not have hearing aids. The proportion of underserved patients decreases with the availability of health professionals in the towns.

The proportion of patients with hearing loss was 97%, where hearing loss was defined as a threshold of 30 dB or more in at least one ear and at least one of the frequencies 0.5, 1.0, 2.0 and 4.0 kHz, which is one indicator for qualifying for a hearing aid. In the HörMat study [11], a hearing loss of 77.8% in patients older than 60 years with hearing impairment defined as ≥ 25 dB in one of the frequencies between 0.5 and 4.0 kHz was observed. Lower proportions ranging between 16 and 25%, depending on age and definition of hearing loss, were observed in a recent review [10]. Sohn and Jörgenshaus [15], who included patients aged above 14 years in a primary care practice, diagnosed hearing loss in 19% of their patients with thresholds of ≥ 40 dB for one of the frequencies 0.5, 1.0, 2.0 or 4.0 kHz. In the HörSTAT study [12], patients between 18 and 97 years of age were included (Table 3).

**Table 3:** Overview of definitions of hearing loss and observed proportions in a selection of previous studies in Germany

References	Age (years)	Definition of hearing loss	Proportion (%)
Löhler et al. [11]	60–75	Average threshold of ≥ 25 dB for at least one of the frequencies 0.5, 1, 2 or 4 kHz	77.8
Von Gablenz et al. [12]	18–97	Average threshold of > 25 dB for the frequencies 0.5, 1, 2 and 4 kHz	15.7
Robert Koch Institut [13]	> 18	Subjective self-assessment	Minor difficulties: 18.8 major difficulties: 2.7
Neubauer and Gmeiner [14]	19– ≥ 90	According to ICD-10	~ 10% over age 70; up to 15% over 80 years of age
Sohn and Jörgenshaus [15]	> 14	Average threshold of ≥ 40 dB for at least one of the frequencies 0.5, 1, 2, 3 or 4 kHz	19
This study	37–93	Average threshold of ≥ 30 dB for at least one of the frequencies 0.5, 1, 2 and 4 kHz in at least one ear	97

The preceding summary illustrates the heterogeneity of definitions for hearing loss used in different studies, which complicates the interpretation of corresponding proportions. We chose our definition, because it is used by German health insurances for the indication of a hearing aid. The study with the most similar definition of hearing loss [11], i.e., ≥ 25 dB instead of ≥ 30 dB, also observed a high proportion of patients with of hearing loss (77.8%), albeit lower than the 97% observed in our study. An explanation could be older age and possible self-selection in our study. If we apply the 25 dB threshold used in the HörMat study [11] to our data, the proportion of patients with hearing loss is 98% (95% CI 97–100%). The proportion of disabling hearing loss according to the World Health Organization, i.e., more than an average of 40 dB for frequencies 0.5, 1, 2 and 4 kHz, is 59% (95% CI 52–66%) in our data (https://www.who.int/pbd/deafness/estimates/en/, accessed Dec 17, 2020). Studies with substantially lower proportions of hearing loss either based their definition on the average over four frequencies rather than at least one frequency [12], used subjective criteria [13, 14], or used substantially higher thresholds (e.g., ≥ 40 dB [15]). Therefore, the observed proportion of patients with hearing loss in our study is indeed very high compared to other studies but is not per se inconsistent.

The observed proportion of subjects without hearing aids among all subjects with hearing loss greater or equal than 30 dB (77%) was higher compared with two studies identified in the review by Löhler et al. [10]. Von Gablenz et al. [21] reported 5.8% (age 60–69 years), 18.3% (70–79 years) and 32.6% (≥ 80 years) in the HörSTAT study, while Sohn and Jörgenshaus [15] observed that 2% of all participants had a hearing aid while the proportion of patients with of hearing impairment was 16%.

Our data show that untreated hearing loss is common in rural areas and may reach close to 100% in areas with no hearing aid technician and no ENT physician. This is consistent with Chan et al. [22], who observed that distance to hearing healthcare services was associated with the timing of acquisition of hearing aids. Key motivators to seek care include degree of hearing loss, self-efficacy, family support, and self-recognition of hearing loss [23].

The need for optimal rehabilitation of hearing impairments is not only important for optimal sound transmission to the auditory cortex. In addition, it is known that the auditory impulse on vestibulospinal reactions is an important component in balance regulation [24]. Dawes et al. [25] observed an association between hearing aid use and better cognition. Also, hearing aid use is associated with delayed diagnosis of dementia, depression and anxiety [26].

Our preliminary study includes only a small number of self-selected participants. It is possible that the observed proportion of hearing loss is an overestimate, since hearing-impaired persons may have been particularly attracted by the offer of a cost-free audiometry test. Moreover, only limited demographic and other patient-specific information was recorded, and was entirely missing for a fraction of the subjects. However, our study has several strengths. Audiometry was performed by ENT physicians on standardized equipment in a vehicle which was specifically noise-insulated for this purpose. The high proportion of hearing-impaired persons allowed us to estimate the percentage of underserved patients with high precision (77%, 95% CI 69–84). The knowledge about the availability of hearing aid technicians and/or ENT physicians in the selected areas enabled us to evaluate determinants of underserving in Germany.

Although our study took place in a local context, the relevance of the objective goes far beyond the particular region studied. Thinly populated areas are common in all European countries (on average 30% of the area), and population density is known to be inversely correlated with health in industrialized countries [20]. The patterns observed here may, therefore, generalize to substantial proportions of the population in countries such as Germany, France, the Netherlands, Spain, and the UK.

Hearing loss can have profound effects not only on interpersonal communication, but also on health, independence, wellbeing, quality of life, and daily function. It is a field in which modest interventions have the potential of producing a substantial reduction in the global burden of disease. Large representative studies are needed, particularly in rural areas, to assess the level of hearing loss, the fraction of persons without adequate help and the reasons for this, so that interventions, such as mobile diagnostic units, can be designed and evaluated. These interventions aim to improve the hearing of the population to increase their quality of life and to prevent severe morbidity known to be associated with untreated hearing loss, such as falls, dementia, depression, and other diseases.

## Data Availability

The data are available upon reasonable request to the corresponding author.

## Code availability

The STATA code for the analysis of the data is available upon reasonable request to the corresponding author.
